# Safety and Efficacy of Surgery for Metastatic Tumor to the Pancreas: A Single-Center Experience

**DOI:** 10.3390/jcm12031171

**Published:** 2023-02-01

**Authors:** Lucia Moletta, Alberto Friziero, Simone Serafini, Valeria Grillo, Elisa Sefora Pierobon, Giovanni Capovilla, Michele Valmasoni, Cosimo Sperti

**Affiliations:** Department of Surgery, Oncology and Gastroenterology, 1st Surgical Clinic, University of Padua, Via Giustiniani 2, 35128 Padua, Italy

**Keywords:** pancreatic metastases, pancreatic secondary tumors, pancreatectomy, metastasis, renal cell carcinoma

## Abstract

Pancreatic metastases from other neoplasms are rare. The role of surgery for this clinical entity is unclear. The aim of this study was to investigate the role of resection in patients with pancreatic secondary lesions. We observed 44 patients with pancreatic metastases from other tumors. Renal cell carcinoma was the most common primary tumor (*n* = 19, 43.2%). Thirty-seven patients underwent surgery, and pancreatic resection with curative intent was feasible in 35 cases. Fifteen patients (43.2%) experienced major postoperative complications (Clavien-Dindo > 2), and postoperative mortality rate was 5.4%. The median overall survival and disease-free survival were 38 (range 0–186) and 11 (range 0–186) months, respectively. Overall survival and disease-free survival were significantly longer for pancreatic metastases from renal cell carcinoma when compared to other primary tumors. Multivariate analysis confirmed a pathological diagnosis of metastasis from RCC as an independent prognostic factor for overall survival (OR 2.48; 95% CI, 1.00–6.14; *p* = 0.05). In conclusion, radical resection of metastases to the pancreas is feasible and safe, and may confer a survival benefit for selected patients. There is a clear benefit of metastasectomy in terms of patient survival for metastases from renal cell carcinoma, while for those with other primary tumors, surgery seems to be mainly palliative.

## 1. Introduction

Pancreatic metastases (PM) from other tumors are rare, accounting for only about 1–2% of all pancreatic malignancies [1,2,3]. Pancreatic secondary lesions have been described in several autoptic series, the most frequent sites of primary tumors being the kidney, breast, colon-rectum, melanoma and lung [4]. PM are often asymptomatic and diagnosed during routine follow-up after a primary tumor has been resected or detected incidentally on imaging performed for unrelated reasons. Even when there are symptoms, they are usually non-specific, and the differential diagnosis between primary pancreatic tumors and metastases can be challenging [5]. In addition to the lack of a specific clinical presentation, the interval elapsing between the onset of primary tumor and that of pancreatic metastases can vary considerably. This is particularly true for PM from renal cell carcinoma (mRCC), which can be detected several years, and even a decade or more in some cases, after the primary tumor was resected [6]. As well as posing these diagnostic difficulties, PM also represent a therapeutic challenge. Most patients already have widespread disease at diagnosis, while the finding of an isolated pancreatic lesion is far less common. This means that pancreatic resection is rarely indicated for PM. More and more studies have reported successful pancreatic resection for PM in recent years, as lower morbidity and mortality rates after pancreatic surgery at high-volume centers have led to an extension of the surgical indication for benign/borderline lesions, and for PM as well. That said, while surgery is an established procedure for lung and liver metastases in selected patients, the role of pancreatectomy for PM remains to be established [7,8]. The aim of this study was to investigate the safety and efficacy of resection in patients with pancreatic secondary lesions.

## 2. Materials and Methods

We retrospectively reviewed all patients referred to us with PM between January 2000 and December 2019 and included in a prospectively-collected database. During this time interval, we observed 44 patients with PM and among these, 37 received surgery. As a matter of fact, among 522 pancreatic resections performed at our center for malignant lesions, 37 involved patients with PM (7%). All the patients considered here had a histological diagnosis of PM, obtained using FNAC during EUS or a percutaneous needle biopsy or histological examination of surgical specimens after resection. Patients with Von Hippel Lindau disease were not considered because of the different biology and behavior of renal tumors in these patients. Patients with primary pancreatic tumors and those with direct pancreatic invasion by other intra-abdominal tumors were excluded as well. The following data were collected and analyzed: basic demographics, characteristics of primary tumor, interval between onset of primary tumor and secondary pancreatic lesion, presenting symptoms, radiological and laboratory tests, presence of extrapancreatic disease, patients’ performance status, surgical strategy and approach, morbidity, mortality, length of ICU and hospital stay, details of neoadjuvant and adjuvant treatments, follow-up, disease-free survival, and overall survival. The presence of extrapancreatic disease was defined as the presence of metastatic foci other than in the pancreas, not as local extensions of the PM. In cases of metachronous lesions, the disease-free interval was defined as the time elapsing between resection of the primary tumor and the detection of PM. Patients’ comorbidities and fitness before surgery were assessed according to the Charlson Comorbidity Index (CCI), and the American Society of Anesthesiologists classification, respectively [9].

### 2.1. Surgical Approach

A pylorus-preserving pancreaticoduodenectomy (PPPD) or a Whipple procedure was performed in patients with pancreatic head lesions. A duodenum-preserving pancreatic head resection (DPPHR) was performed in cases of small single pancreatic head lesions not involving the duodenum. Distal pancreatectomy (DP) with or without splenectomy was the procedure of choice for lesions of the body/tail. Central pancreatectomy (CP) was performed for single lesions of the pancreatic body. Total pancreatectomy was reserved for patients with multiple PM or microscopic tumor infiltration of the resection margin on intraoperative frozen section examination. Histological reports on surgical specimens were reviewed, paying particular attention to resection margins and lymph node status. Postoperative complications were assessed using the Clavien-Dindo classification [7]. Postoperative pancreatic fistula, hemorrhage and delayed gastric emptying were defined and assessed according to the International Study Group of Pancreatic Surgery (ISGPS) classification [10,11,12]. Postoperative mortality was defined as death occurring during the postoperative hospital stay or within 30 days after the surgical procedure.

### 2.2. Postoperative Follow-Up

After pancreatic resection, all patients were followed up every three months for the first two years, and then every six months for five years. The follow-up involved a clinical examination, laboratory tests, CT scans of the chest and abdomen, and 18-fluorodeoxy-glucose positron emission tomography (18-FDG-PET), when indicated. We defined a tumor recurrence as the appearance of new metastatic foci on follow-up radiological examinations, with or without histological confirmation. Overall survival was calculated from the date of the pancreatic resection to the latest available follow-up. The time of disease recurrence was recorded as the date of the first radiological detection of tumor relapse during the postoperative follow-up.

### 2.3. Statistical Analysis

Data are expressed as medians (interquartile range—IQR) or means (±standard deviation—SD) for quantitative variables, and as absolute frequencies (percentages) for categorical variables. Quantitative variables were compared using the Mann-Whitney test or the Kruskal-Wallis test, as appropriate. Categorical variables were analyzed with the Chi-square test or Fisher’s exact test in the case of absolute frequencies < 5. We considered statistical test findings significant for *p*-values of less than 0.05. *p*-values for multiple comparisons were corrected for alpha inflation using the Bonferroni method. Overall and recurrence-free survival estimates were calculated using the Kaplan-Meier method. The survival curves were compared using the log-rank test or the Gehan-Breslow-Wilcoxon test, as appropriate. Univariate and multivariate analyses were run to identify potential risk factors for overall survival. Variables showing a *p*-value < 0.1 on univariate analysis were included in the multivariate logistic regression to identify independently associated risk factors. We performed the statistical analysis using STATA, version 14.1 (StataCorp LLC, College Station, TX, USA).

## 3. Results

### 3.1. Demographics and Clinical Presentation

Forty-four patients were referred to our Center for PM (M/F 22/22; median age 66 years, range 41–78). Their demographics are summarized in Table 1 and Table 2. Renal cell carcinoma was the most common primary tumor (*n* = 19, 43.2%), followed by colorectal cancer (*n* = 12, 27.2%), sarcoma (*n* = 4, 9.1%), melanoma (*n* = 4, 9.1%), lung cancer (*n* = 2, 4.5%), schwannoma (*n* = 1, 2.3%), gastric cancer (*n* = 1, 2.3%), and small bowel carcinoid tumor (*n* = 1, 2.3%). The pancreas was the first site of metastases in most patients (*n* = 38, 86.4%). The pancreatic lesion was synchronous with the primary tumor in 11 cases. Among patients with metachronous metastases, the median time elapsing between resection of the primary tumor and the detection of PM was 72 months (range 9–260). Nearly half of the patients (*n* = 21, 47.7%) were symptomatic when their PM was diagnosed. Abdominal pain (*n* = 9) and obstructive jaundice (*n* = 8) were the most common symptoms, followed by fatigue, weight loss, vomiting, upper gastrointestinal hemorrhage, and carcinoid syndrome.

### 3.2. Diagnostic Work-Up

**Figure 1:**
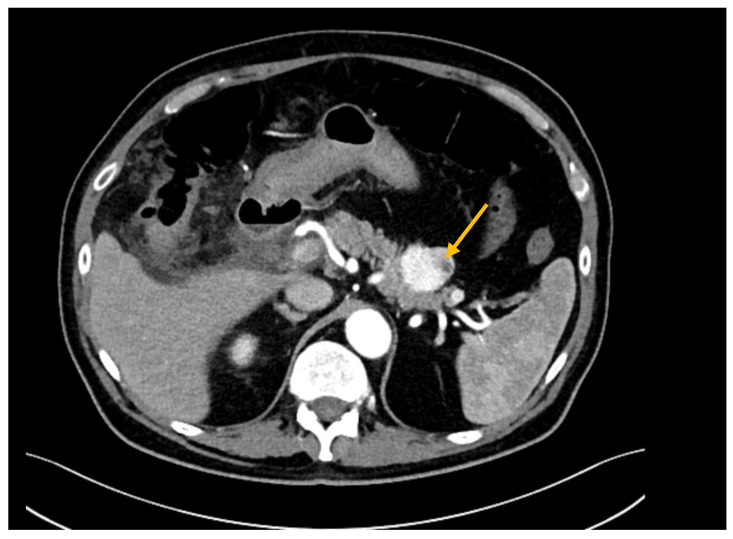
CT scan showing a hypervascular lesion of the pancreatic tail (PM from RCC, yellow arrow).

**Figure 2:**
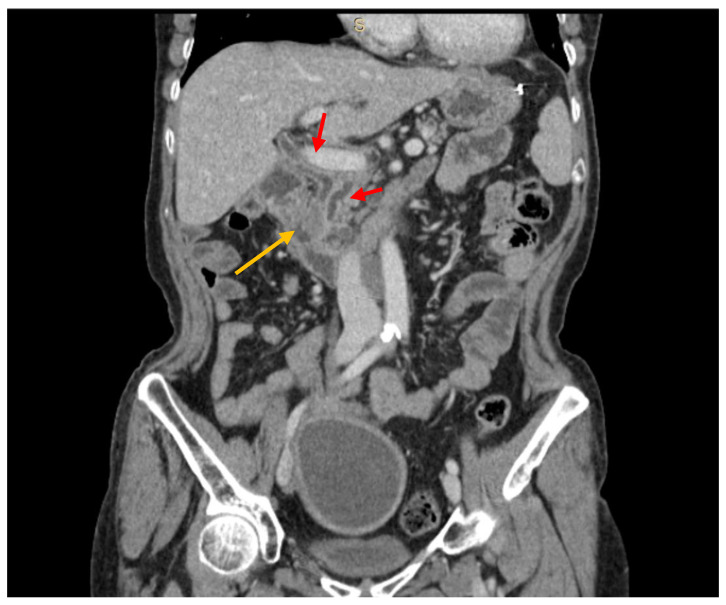
CT scan showing a hypovascular lesion (yellow arrow) of the pancreatic head, causing both bile duct and panceatic duct dilation (red arrows) (PM from colon cancer).

The diagnostic work-up included CT scans in 36 cases (82%), MRI in 18 (40.9%), both CT scans and MRI in 10 (22.7%), and PET-CT in 23 (52.3%) (Figure 1 and Figure 2). Histological confirmation was obtained from EUS-FNAC (*n* = 5, 11.4%), surgical specimens (*n* = 37, 84.1%), or percutaneous needle biopsy (*n* = 2, 4.5%). EUS-FNAC detected PM correctly in three cases, while the material retrieved was insufficient in one, and in one patient FNAC detected the presence of cancer but was unable to distinguish between pancreatic carcinoma and PM. Twenty-nine patients had a single metastasis while 15 had 3 pancreatic lesions on average (range 2–7). Six patients (13.6%) had a previous history of metastases (treated with surgery in five cases) and 15 patients (34.1%) presented with extra-pancreatic disease at the time of diagnosis of their PM. The main sites of extra-pancreatic disease were: liver (*n* = 5), lung (*n* = 5), colon (*n* = 5), and peritoneum (*n* = 1). Compared with patients who had other primary tumors, those with metastases from RCC experienced a longer disease-free interval between their primary tumor and the onset of PM, and they more often presented with multiple PM.

### 3.3. Surgical Approach and Postoperative Outcomes

Thirty-seven patients underwent surgery, and pancreatic resection with curative intent was feasible in 35 cases. Details of the surgical resections and postoperative outcomes are given in Table 3. Only two patients received neoadjuvant treatment: one with PM from colon cancer underwent preoperative FOLFIRI; and one with PM from sarcoma was given preoperative gemcitabine plus dacarbazine. Radical resection of PM was not feasible in two patients due to local involvement of the mesenteric vessels. These two patients underwent palliative procedures (hepaticojejunostomy for jaundice, and gastrojejunostomy for duodenal stenosis). Standard oncological resections were performed in 30 patients (13 pancreaticoduodenectomies [nine PPPD and four Whipple procedures], 16 distal pancreatectomies, and one total pancreatectomy), while a limited pancreatic resection was performed in 5 (spleen-preserving distal pancreatectomy in two cases, central pancreatectomies in two, and DPPHR in one). Only one patient had a laparoscopic procedure (laparoscopic distal resection); all other patients underwent open surgery. Sixteen of the patients who had a pancreatic resection also underwent an associated procedure. In six patients, the PM was synchronous with the primary tumor, which was resected at the same time (four colectomies, one radical nephrectomy, and one ileal resection). In four patients, a multi-visceral resection was needed due to other metastases (one liver wedge resection, one lung wedge resection, one left hemicolectomy, one left adrenalectomy). In two patients, the local extension of the pancreatic disease demanded an associated resection (a left nephrectomy and adrenalectomy in both cases). Finally, associated procedures were performed for non-oncological reasons in four cases: a cholecystectomy for chronic cholecystitis; an adhesiolysis and ileal resection for intraoperative iatrogenic small bowel injury and two partial colectomies for intestinal ischemia.

The median length of hospital stay was 13 days (range 7–89). Fifteen patients (43.2%) experienced major postoperative complications (Clavien-Dindo > 2). Eight (21.6%) developed a postoperative grade B pancreatic fistula. Other complications included: two biliary leaks, one enteric leak, one small bowel obstruction due to postoperative adhesions, one postoperative hemorrhage, one case of pneumonia, one case of sepsis due to methicillin-resistant staphylococcus aureus (MRSA). Surgical reintervention was required in five patients. Two patients (5.4%) died after surgery, one due to pulmonary embolism, the other as a result of a massive gastrointestinal hemorrhage. Six patients (17.1%) developed postoperative diabetes. Seventeen (94.4%) of the 18 patients who were symptomatic before surgery experienced the resolution of their symptoms afterwards. Histology showed peripancreatic lymph node involvement in 13 patients. Notably, none of the resected specimens of mRCC in this case series revealed peripancreatic lymph node metastases.

Among the seven patients who did not receive a surgical approach, two refused surgery, while the other five cases had either a metastatic spread or a locally advanced tumor. Four of them received palliative chemotherapy while one case with PM from RCC was treated with a multi-target receptor tyrosin kinase inhibitor.

### 3.4. Survival Analysis

**Figure 3:**
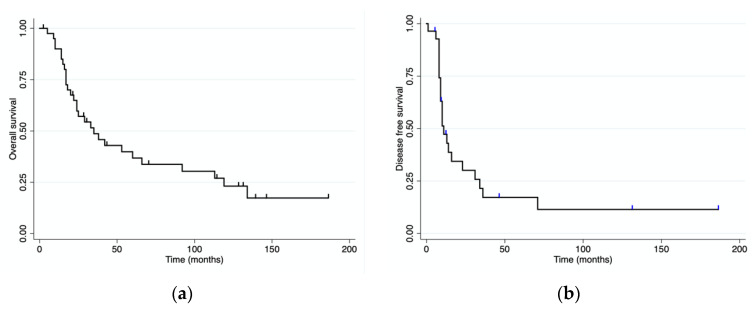
Overall and disease-free survival for the whole cohort of patients. (**a**) Overall survival; (**b**) disease-free survival.

**Figure 4:**
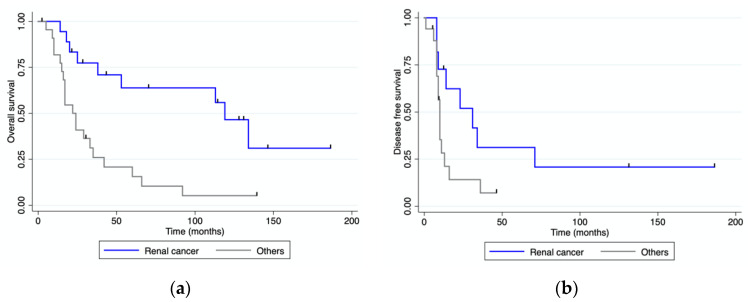
Overall and disease-free survival for patients with mRCC vs. patients with PM from other primary tumors. (**a**) Overall survival; (**b**) disease-free survival.

**Figure 5:**
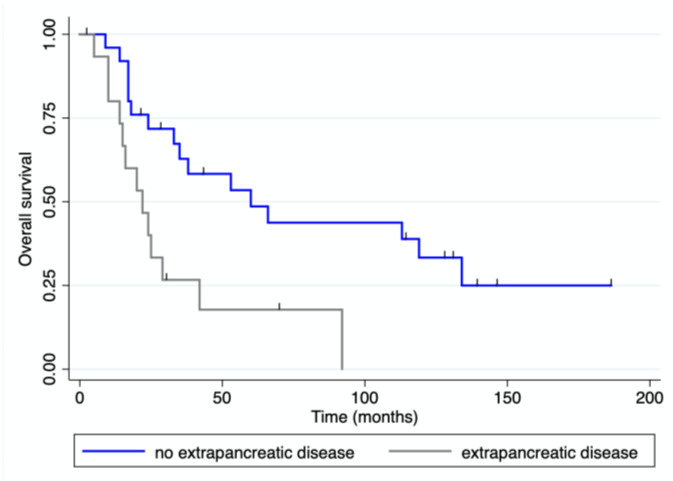
OS for patients presenting with vs. without extrapancreatic disease.

**Figure 6:**
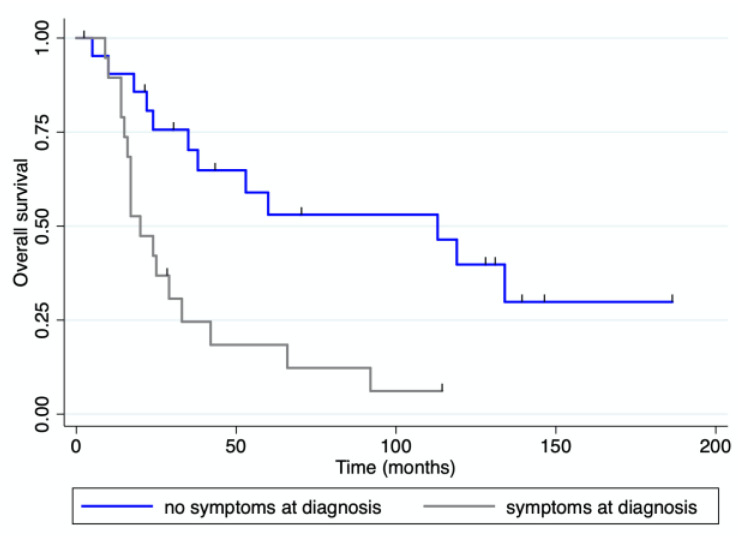
OS for patients divided by presence/absence of symptoms at diagnosis of PM.

With a median follow-up of 33 months (range 0–186) after pancreatic resection, four patients were alive and disease-free, and eight patients were alive with recurrent disease. Eighteen patients received adjuvant treatment after their pancreatic resection. Twenty-seven patients died of progressive disease and two died from unrelated causes. The median overall and disease-free survival for the whole cohort of patients were 38 (range 0–186) and 11 (range 0–186) months, respectively (Figure 3). The median overall survival for patients with mRCC was 119 months (range), while it was 15.5 months (range 0–60) for patients with colorectal PM, 36 months (range 14–66) for those with PM from sarcoma, and 20 months (range 2–24) for those with PM from melanoma. OS and DFS were both significantly longer for patients with mRCC than for those with PM from other primary tumors (Figure 4). Patients presenting at diagnosis with no extrapancreatic disease or symptoms had a longer OS than those with extrapancreatic foci of disease and symptoms at diagnosis (Figure 5 and Figure 6). No differences in OS were noted between patients not given adjuvant treatment after pancreatic resection and those given post-surgical chemotherapy.

Univariate analysis (Table 4) identified two risk factors associated with survival: symptoms at diagnosis and a pathological diagnosis of metastasis from RCC. Multivariate Cox proportional hazards regression analysis confirmed a pathological diagnosis of metastasis from RCC as an independent prognostic factor for overall survival (HR 2.48; 95% CI, 1.00–6.14; *p* = 0.05).

## 4. Discussion

Pancreatic secondary tumors are rare [13]. Their clinical presentation is often non-specific and they tend to be detected during the follow-up performed after resection of the primary tumor. In our case series, almost half of the patients presented with symptoms at diagnosis of PM, while the remainder were diagnosed with PM during their routine follow-up after surgery for a primary tumor. Our findings are consistent with previous reports (a recent systematic review by Huang et al. [14] found that 50% of patients with PM were asymptomatic at the time of diagnosis) and confirm the importance of routine follow-up in this setting. This is particularly true for patients with mRCC who may develop PM a long time after being treated for their primary tumor [15]. Fullarton and Burgoyne reported intervals between nephrectomy and the detection of PM spanning as long as 27 years [16]. In our cohort, the longest interval was 21 years after the resection of a primary RCC, and the median interval was about nine years (range: 0–260 months). Long-term follow-up is therefore strongly recommended in such patients, even for those found recurrence-free for more than five years. CT scans seem to be the most effective imaging modality for identifying PM [14]. They also provide information on vascular relationships, possible multiple PM, and any presence of other metastatic foci. It is sometimes difficult to distinguish other subtypes of metastatic lesions from pancreatic adenocarcinoma; as a matter of fact, mRCC has a typically hyper-vascularized appearance on cross-sectional imaging [17], while other types of PM might look hypo-vascular, resembling primary adenocarcinoma.

EUS-FNAC has been recommended for obtaining biopsies in cases of PM because of its diagnostic efficacy and safety profile [18,19]. Smith et al. [19] reported on 22 patients whose EUS- FNAC correctly revealed the presence of a pancreatic secondary lesion. On the other hand, Lee et al. [20] found that the FNAC failed to provide a definitive diagnosis for 53% of patients ultimately confirmed by the pathologist as cases of PM. Only a minority of our patients underwent a preoperative biopsy (*n* = 7), which proved diagnostically accurate only in three of them. A preoperative histological assessment was only obtained for cases of PM not amenable to surgical resection, while it was omitted in surgical candidates.

The role of surgery in patients with PM remains to be established. In recent years, several authors have reported acceptable rates of morbidity and mortality following pancreatic resection for PM. In our case series, the postoperative morbidity rate was 40.5%, with eight patients experiencing clinically relevant postoperative pancreatic fistula. The in-hospital mortality rate was 5.4%. Lee et al. [20] reported comparable postoperative morbidity and mortality rates of 56% and 3.1%, respectively, among 97 patients undergoing pancreatic resection for PM. These figures are similar to the postoperative morbidity and mortality rates reported for pancreatectomies performed for pancreatic adenocarcinoma, indicating acceptable short-term outcomes in this setting [21].

The type of surgical procedure is another controversial aspect in the treatment of PM. Some authors have proposed ‘atypical’ resections such as enucleation to reduce the morbidity of surgical treatment [6], but Bassi et al. reported a 29% rate of pancreatic recurrences after performing atypical resections in cases of metastases from RCC [22]. It is unclear whether such recurrences after atypical resections are due to an inadequate surgical procedure or to undetected multifocality. Multiple lesions are not rare, particularly in patients with metastases from RCC. One third of our patients presented with multiple PM, and more than half of cases with mRCC had multiple pancreatic lesions. Thus, it would seem wise to check carefully for multiple pancreatic lesions, regardless of the choice of surgical approach. Ultimately, the choice between standard and atypical resections is probably unimportant, providing the resection margins obtained are cancer-free.

Another controversial point concerns the need for regional lymph node dissection along with pancreatic resections, particularly for patients with mRCC, since several reviews reported finding no lymph node involvement in such cases. Faure et al. detected no lymph node metastases in their patients and consequently deemed lymph node dissection optional [3]. Law et al. found one patient (7%) with a regional peripancreatic lymph node metastasis, however, and therefore recommended standard oncological resection [23]. None of our patients undergoing pancreatic resection for PM from RCC had any positive lymph nodes. An individualized approach should probably be taken, an optimal resection strategy providing for lymph node dissection in the event of nodal involvement being suspected or detected pre- or intra-operatively.

Long-term outcomes seem to relate strictly to the tumor’s biology. Several authors found a survival benefit for patients with PM from RCC treated with surgery. [24] mRCC has a unique biology with a slow progression and lengthy disease-free interval. The median survival of patients with mRCC in our series was nearly 10 years, and their OS was significantly longer when compared to patients with PM from other primary tumors. Milanetto et al. [25] reported a median OS of over 11 years in a cohort of 39 patients with PM from RCC who underwent surgical resection; the cohort’s 1-, 3-, 5-, and 10-year OS rates were 94%, 88%, 79%, and 55%, respectively. Other authors [26,27] reported a significantly longer five-year survival in patients with PM from RCC who underwent surgery (72–73%) than in those who did not (0–14%). More recently, several angiogenic agents have been used for the treatment of mRCC with promising results [28]. Santoni et al. conducted a multicenter study [29] on long-term outcomes in patients with PM from RCC comparing those undergoing surgery with those given tyrosine-kinase inhibitors. The results achieved by the two approaches were similar, but surgical resection was associated with a higher percentage of patients remaining disease-free and a longer disease-free survival. Surgery seems a reasonable option for patients with PM from RCC, given the favorable long-term outcomes reported in the literature. A multidisciplinary approach is advisable, however, and further studies are needed to establish how best to combine surgery with medical treatment in the various phases of the disease.

The role of metastasectomy for PM from other tumors is less clear. There are fewer published reports on the outcomes of pancreatic surgery in this setting, but PM from other primary tumors do not appear to have the same favorable outcomes as PM from RCC. The other primary tumors involved in PM have a more aggressive nature and shorter median survival rates, making pancreatic resection more debatable. In our sample, the OS was 15.5 months (range 0–60) for colorectal PM, 36 months (range 14–66) for PM from sarcoma, and 20 months (range 2–24) for PM from melanoma. On the other hand, although these patients’ OS was not comparable with that of patients resected for mRCC, all symptomatic cases who underwent surgery experienced a relief of their preoperative symptoms. A more cautious patient selection is therefore recommended for PM from other tumors. Resection of such metastases seems to have mainly a palliative role, to provide symptom relief, although surgical resection might be advocated in selected cases as part of a multimodality treatment of metastatic colorectal cancer, sarcoma, and melanoma.

This study has several limitations, particularly its retrospective design and small number of patients. Moreover, the treatment of many tumors has been changed during this long period of time. However, the rarity of presentation inevitably limits the sample size and the surgical treatment of pancreatic metastases remained substantially the same during the study period.

## 5. Conclusions

In conclusion, radical resection of metastases to the pancreas is feasible and safe, and may confer a survival benefit for selected patients. The usefulness of pancreatic resection depends mainly on the biology of the primary tumor metastasizing to the pancreas. There is a clear benefit of metastasectomy in terms of patient survival for metastases from RCC, while for those from other primary tumor surgery metastasectomy seems to be mainly palliative. A case-by-case, multidisciplinary assessment is recommended, as surgery is part of the multimodality treatment of PM. Further studies are needed to establish how best to combine surgery with medical treatments for the different metastatic diseases of the pancreas.

## Figures and Tables

**Table 1:** Patients’ demographics and characteristics of their pancreatic metastases.

		**All (*n* = 44)**		**RCC (*n* = 19)**		**Other Tumors (*n* = 25)**		***p***
		***n***	**%**	***n***	**%**	***n***	**%**	
**Sex**	**M**	22	50	11	57.9	11	44.0	0.27
	**F**	22	50	8	42.1	14	56.0	
**Age** mean ± SD, years		63.7 ± 10		66.4 ± 8.1		61.7 ± 10.7		0.06
**Preoperative diabetes**		6	13.6	4	21.0	2	8.0	0.21
**ECOG > 1**		4	9.1	2	10.5	2	8.0	0.59
**Symptoms at diagnosis**		21	47.7	6	31.6	15	60.0	0.6
**Interval from primary tumor to PM** median (range), months		48 (0–260)		111 (0–260)		29 (0–115)		**0.009**
**Previous metastases**		6	13.6	2	10.5	4	16.0	0.48
**Synchronous**		11	25	3	15.8	8	32.0	0.19
**Multiple pancreatic lesions**		15	34.1	12	63.1	3	12.0	**0.001**
**Extrapancreatic disease**		15	34.1	3	15.8	12	48.0	**0.001**
**Location of PM**								
Head		13	29.5	3	15.8	11	44.0	**0.04**
Body		7	15.9	2	10.5	4	16.0	0.48
Tail		9	20.4	2	10.5	7	15.9	0.15
Multiple locations		15	34.1	12	63.1	3	12.0	**0.001**
**Size of largest PM** Median (range), mm		30.0 (7.0–100.0)		25.0 (7.0–40.0)		30.0 (20.0–100.0)		**0.0124**

Statistical analysis: Categorical variables were analyzed with Chi square test, with the exception of variables with absolute frequencies <5 which were analyzed with Fisher exact test (i.e., preoperative diabetes). Quantitative variables (once verified that they are not normally distributed) were compared with Mann-Whitney test. Bold values indicates statistical significance.

**Table 2:** Characteristics of primary tumors in patients with mRCC.

Variable	*n* (%)
**Side**	Right kidney: 8 (42.1) Left kidney: 10 (52.7) Bilateral: 1 (5.2)
**Histology**	Clear cell carcinoma: 19 (100)
**Fuhrman nuclear grade**	G1: 2 (10.5) G2: 14 (73.7) G3: 3 (15.8)
**TNM**	Stage 1: 4 (21.1) Stage 2: 5 (26.3) Stage 3: 8 (42.1) Stage 4: 2 (10.5)
Type of intervention	Unilateral radical nephrectomy 18 (94.7) Left radical nephrectomy + right partial nephrectomy 1 (5.3)
**Timing** of PM from RCC, *n*	Synchronous: 3 (15.8)
	Metachronous: 16 (84.2)
**Diagnostic work-up**	
18-FDG-PET	Performed in 7, positive in 5
Octreoscan	Performed in 4, positive in 2
CT scan	Performed in all patients, all positive

**Table 3:** Postoperative outcomes in patients undergoing surgery for PM.

Variables	Total	RCC	Other Tumors	*p*
**Surgical procedures** *n* (%)	37	16	21	
PD	13 (35.1)	1 (6.2)	12 (57.1)	**0.001**
DP	18 (48.6)	9 (56.2)	9 (42.9)	0.32
CP	2 (5.4)	2 (12.6)	0	0.18
DPPHR	1 (2.7)	1 (6.2)	0	0.43
TP	1 (2.7)	1 (6.2)	0	0.43
Exploratory/palliative	2 (5.4)	2 (12.6)	0	0.18
**Operating time** min, median (range)	208.0 (115.0–470.0)	242.5 (115.0–395.0)	300.0 (165.0–470.0)	0.05
**Associated procedures** *n* (%)	16 (43.2)	2 (12.5)	14 (66.7)	**0.001**
**R0 resection** *n* (%)	29 (78.4)	12 (75)	17 (80.9)	0.48
**Lymph node metastases** *n* (%)	13 (35.1)	0 * (0)	13 (61.9)	**<0.001**
**LOS** median (range)	13 (7–89)	10 (7–66)	17 (7–89)	0.09
**Major postoperative complications** *n* (%)	15 (40.5)	7 (43.7)	8 (38.1)	0.50
**Pancreatic fistula** *n* (%)				
**B**	8 (21.6)	6 (37.5)	2 (9.5)	**0.05**
**C**	0 (0)	0 (0)	0 (0)	1.00
**In-hospital mortality** *n* (%)	2 (5.4)	1 (6.2)	1 (4.7)	0.69
**Postoperative diabetes** *n* (%)	5 (13.5)	3 (18.7)	2 (9.5)	0.37
**Adjuvant treatment** *n* (%)	18 (48.6)	7 (43.7)	11 (52.4)	0.43
**Disease recurrence** *n* (%)	25 (67.6)	9 (56.2)	16 (76.2)	0.18
**Disease-free survival** median (range)	11 mo (0–186)	31 mo (0–186)	10 mo (0–46)	**0.04**
**Overall survival** median (range)	38 mo (0–186)	119 mo (0–186)	24 mo (0–139)	**<0.001**

PD = pancreaticoduodenectomy, DP = distal pancreatectomy, CP = central pancreatectomy, DPPHR = duodenum-preserving pancreatic head resection, TP = total pancreatectomy, LOS = length of stay, mo = months. The PD included 11 ppPD and 2 Whipple. The DP included 2 spleen-preserving DP, associated with enucleation in one case. * 1 data missing. Statistical analysis: categorical variables were analyzed with Chi square test, with the exception of variables with absolute frequencies <5, which were analyzed with Fisher exact test. Quantitative variables (once it was verified that they were not normally distributed) were compared with Mann-Whitney test. Bold values indicates statistical significance.

**Table 4:** Univariate and multivariate analysis for prognostic factors influencing survival. Bold values indicates statistical significance; ns, not significant.

Variables		Univariate	Multivariate	
	*n* (%)	*p*	OR (95% CI)	*p*
Synchronous PM	11 (25)	ns	-	-
Renal cell carcinoma	19 (43.2)	**0.001**	2.48 (1.00–6.14)	**0.050**
Symptoms	21 (47.7)	**0.003**	2.08 (0.86–5.00)	0.101
Postoperative complications	15 (40.5)	ns	-	-
Adjuvant treatment	18 (48.6)	ns	-	-

## Data Availability

The data presented in this study are available on request from the corresponding author.
